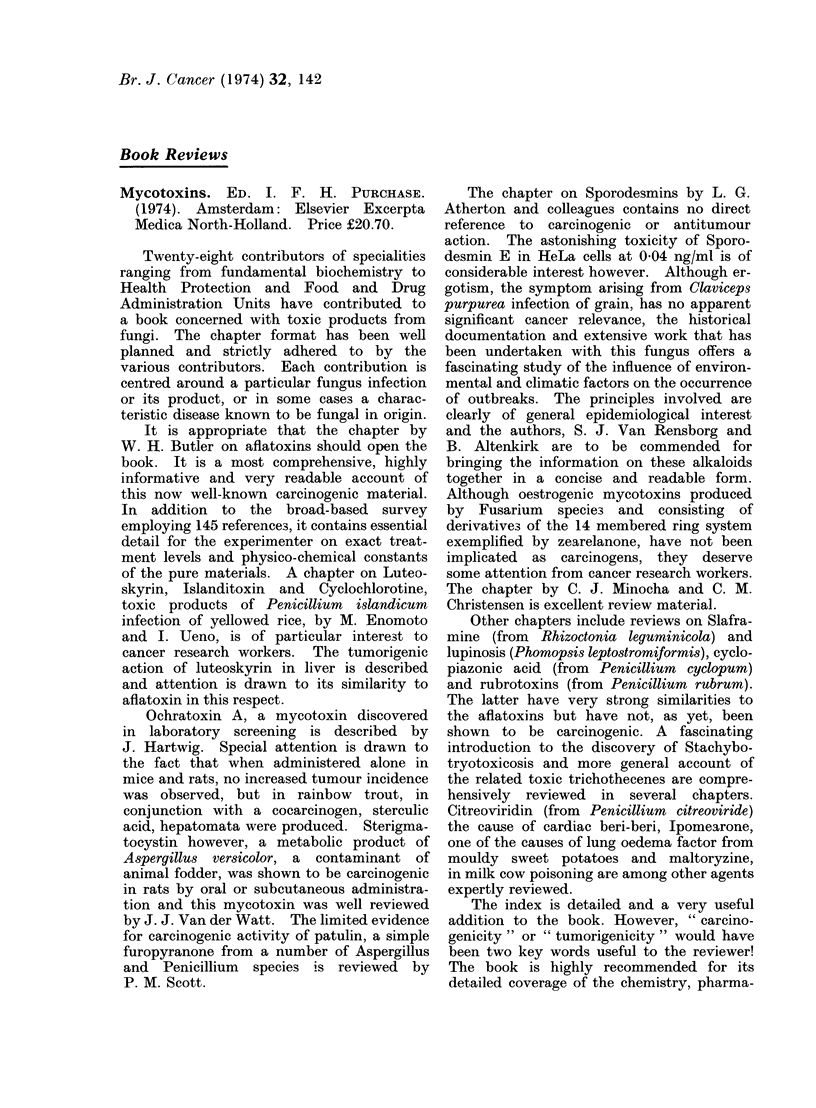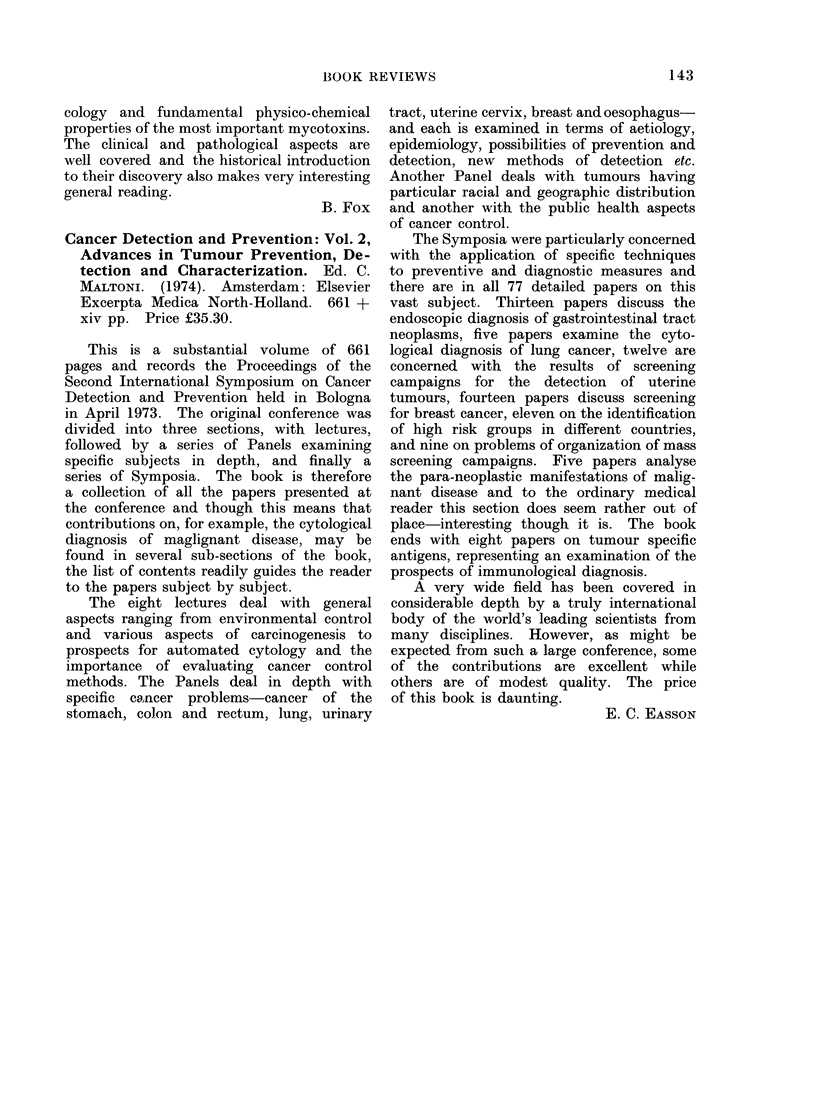# Mycotoxins

**Published:** 1975-07

**Authors:** B. Fox


*Br. J. Cancer* (1974) **32**, 142

### *Book Reviews*

**Mycotoxins.** Ed. I. F. H. Purchase. (1974). Amsterdam: Elsevier Excerpta Medica North-Holland. Price £20.70.

Twenty-eight contributors of specialities ranging from fundamental biochemistry to Health Protection and Food and Drug Administration Units have contributed to a book concerned with toxic products from fungi. The chapter format has been well planned and strictly adhered to by the various contributors. Each contribution is centred around a particular fungus infection or its product, or in some cases a characteristic disease known to be fungal in origin.

It is appropriate that the chapter by W. H. Butler on aflatoxins should open the book. It is a most comprehensive, highly informative and very readable account of this now well-known carcinogenic material. In addition to the broad-based survey employing 145 references, it contains essential detail for the experimenter on exact treatment levels and physico-chemical constants of the pure materials. A chapter on Luteoskyrin, Islanditoxin and Cyclochlorotine, toxic products of *Penicillium islandicum* infection of yellowed rice, by M. Enomoto and I. Ueno, is of particular interest to cancer research workers. The tumorigenic action of luteoskyrin in liver is described and attention is drawn to its similarity to aflatoxin in this respect.

Ochratoxin A, a mycotoxin discovered in laboratory screening is described by J. Hartwig. Special attention is drawn to the fact that when administered alone in mice and rats, no increased tumour incidence was observed, but in rainbow trout, in conjunction with a cocarcinogen, sterculic acid, hepatomata were produced. Sterigmatocystin however, a metabolic product of *Aspergillus versicolor*, a contaminant of animal fodder, was shown to be carcinogenic in rats by oral or subcutaneous administration and this mycotoxin was well reviewed by J. J. Van der Watt. The limited evidence for carcinogenic activity of patulin, a simple furopyranone from a number of Aspergillus and Penicillium species is reviewed by P. M. Scott.

The chapter on Sporodesmins by L. G. Atherton and colleagues contains no direct reference to carcinogenic or antitumour action. The astonishing toxicity of Sporodesmin E in HeLa cells at 0·04 ng/ml is of considerable interest however. Although ergotism, the symptom arising from *Claviceps purpurea* infection of grain, has no apparent significant cancer relevance, the historical documentation and extensive work that has been undertaken with this fungus offers a fascinating study of the influence of environmental and climatic factors on the occurrence of outbreaks. The principles involved are clearly of general epidemiological interest and the authors, S. J. Van Rensborg and B. Altenkirk are to be commended for bringing the information on these alkaloids together in a concise and readable form. Although oestrogenic mycotoxins produced by Fusarium species and consisting of derivatives of the 14 membered ring system exemplified by zearelanone, have not been implicated as carcinogens, they deserve some attention from cancer research workers. The chapter by C. J. Minocha and C. M. Christensen is excellent review material.

Other chapters include reviews on Slaframine (from *Rhizoctonia leguminicola*) and lupinosis (*Phomopsis leptostromiformis*), cyclopiazonic acid (from *Penicillium cyclopum*) and rubrotoxins (from *Penicillium rubrum*). The latter have very strong similarities to the aflatoxins but have not, as yet, been shown to be carcinogenic. A fascinating introduction to the discovery of Stachybotryotoxicosis and more general account of the related toxic trichothecenes are comprehensively reviewed in several chapters. Citreoviridin (from *Penicillium citreoviride*) the cause of cardiac beri-beri, Ipomearone, one of the causes of lung oedema factor from mouldy sweet potatoes and maltoryzine, in milk cow poisoning are among other agents expertly reviewed.

The index is detailed and a very useful addition to the book. However, " carcinogenicity " or " tumorigenicity " would have been two key words useful to the reviewer! The book is highly recommended for its detailed coverage of the chemistry, pharmacology and fundamental physico-chemical properties of the most important mycotoxins. The clinical and pathological aspects are well covered and the historical introduction to their discovery also makes very interesting general reading.

B. Fox